# Reduction of Endoplasmic Reticulum Stress Improves Angiogenic Progenitor Cell function in a Mouse Model of Type 1 Diabetes

**DOI:** 10.1038/s41419-018-0501-5

**Published:** 2018-04-27

**Authors:** Maulasri Bhatta, Krishna Chatpar, Zihua Hu, Joshua J. Wang, Sarah X. Zhang

**Affiliations:** 1 Department of Ophthalmology and Ross Eye Institute, University at Buffalo, State University of New York, Buffalo, NY USA; 2 SUNY Eye Institute, State University of New York, New York, NY USA; 3 Department of Biological Sciences, University at Buffalo, The State University of New York, Buffalo, NY 14260 USA; 4 Center for Computational Research, New York State Center of Excellence in Bioinformatics and Life Sciences, Department of Biostatistics, Department of Medicine, State University of New York at Buffalo, Buffalo, NY 14260 USA; 5 Department of Biochemistry, University at Buffalo, State University of New York, Buffalo, NY USA

## Abstract

Persistent vascular injury and degeneration in diabetes are attributed in part to defective reparatory function of angiogenic cells. Our recent work implicates endoplasmic reticulum (ER) stress in high-glucose-induced bone marrow (BM) progenitor dysfunction. Herein, we investigated the in vivo role of ER stress in angiogenic abnormalities of streptozotocin-induced diabetic mice. Our data demonstrate that ER stress markers and inflammatory gene expression in BM mononuclear cells and hematopoietic progenitor cells increase dynamically with disease progression. Increased CHOP and cleaved caspase­ 3 levels were observed in BM­-derived early outgrowth cells (EOCs) after 3 months of diabetes. Inhibition of ER stress by ex vivo or in vivo chemical chaperone treatment significantly improved the generation and migration of diabetic EOCs while reducing apoptosis of these cells. Chemical chaperone treatment also increased the number of circulating angiogenic cells in peripheral blood, alleviated BM pathology, and enhanced retinal vascular repair following ischemia/reperfusion in diabetic mice. Mechanistically, knockdown of CHOP alleviated high-glucose-induced EOC dysfunction and mitigated apoptosis, suggesting a pivotal role of CHOP in mediating ER stress-associated angiogenic cell injury in diabetes. Together, our study suggests that targeting ER signaling may provide a promising and novel approach to enhancing angiogenic function in diabetes.

## Introduction

Diabetic retinopathy (DR) is a sight-threatening complication of diabetes affecting around 93 million people worldwide^1^. Early clinical features of DR include vascular leakage and focal retinal non-perfusion due to loss of capillaries^2^. Accumulative endothelial injury and failure to repair damaged blood vessels contribute to progressive vascular degeneration and ischemia leading to advanced DR. Upon tissue injury, bone marrow (BM)-derived angiogenic progenitors are released from the BM into circulation and subsequently migrate into injured tissues^3^. In diabetes, this process is hampered, resulting in reduced numbers of circulating angiogenic cells (CACs) in diabetic patients^4^. Furthermore, diabetes disrupts BM homeostasis increasing the production of pro-inflammatory monocytes, which in turn exacerbates retinal inflammation and vascular degeneration^5^.

While the mechanisms underlying the angiogenic abnormalities in diabetes are complex, our recent work suggests that disturbance of endoplasmic reticulum (ER) is potentially involved in diabetic injury of angiogenic progenitors^6^. The ER is one of the major organelles responsible for protein biosynthesis, protein folding and maturation, as well as protein trafficking. Dysfunction of the ER leads to ER stress that activates the unfolded protein response (UPR) to maintain protein homeostasis in normal cells (adaptive UPR) or promote apoptosis of overstressed cells (terminal UPR)^7–10^. In diabetes, increased ER stress is observed in a variety of tissues^11,12^ as well as in angiogenic progenitors^6^. Inhibition of ER stress significantly enhanced the survival and function of angiogenic progenitors cultured in high-glucose (HG) conditions. These findings provide preliminary evidence that ER stress plays a causal role in diabetes-related angiogenic dysfunction.

Herein, we characterized the temporal development of ER stress in BM progenitors and examined the in vivo role of ER stress in angiogenic progenitor dysfunction in a type 1 diabetes model. Our data demonstrate that there is increased ER stress and altered UPR signaling in BM progenitors during diabetes progression. Inhibiting ER stress by chemical chaperone treatment ex vivo or in vivo significantly mitigates diabetes-induced BM pathology, enhances angiogenic progenitor function, and promotes vascular repair in diabetic mice. Knockdown of Chop also improves angiogenic progenitor survival and function. These findings suggest that modulating ER stress may provide a novel approach to improving angiogenic function in diabetes.

## Results

### Decreased numbers of CACs in peripheral blood of diabetic mice

**Fig. 1:**
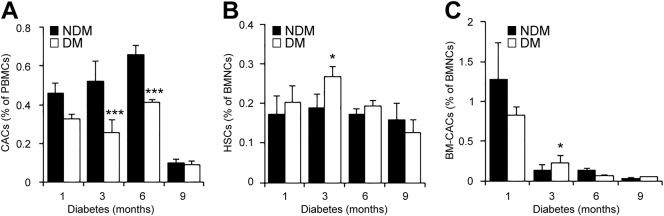
Dynamic changes in levels of bone marrow (BM) progenitor cells and decrease of CACs in diabetic mice. Mononuclear cells from peripheral blood or the BM of diabetic (DM) mice at 1, 3, 6, and 9 months after the onset of diabetes and their respective non-diabetic (NDM) controls were separated by Ficoll gradient centrifugation. **a** Numbers of CACs (Flk-1^+^/Sca-1^+^/CD34^+^) in the peripheral blood. **b** Numbers of BM-HSCs (Lin^−^/c-Kit^+^/Sca-1^+^/CD34^+^). **c** Numbers of BM-CACs (Flk-1^+^/Sca-1^+^/CD34^+^; *n* = 3–6 mice per group). All data are presented as mean ± SD. *P < 0.05, ***P < 0.001 vs. age-matched NDM control mice (two-way ANOVA with bonferroni post hoc test)

Studies have shown that CAC levels in peripheral blood are reduced in patients with type 1 and type 2 diabetes^4,13,14^ as well as in diabetic mice^15,16^. However, a comprehensive analysis of CAC dynamics through the stages of diabetes is lacking. Herein, we examined the CAC levels in peripheral blood of mice with acute (1–3 months) and chronic (6–9 months) diabetes. Prior studies have demonstrated BM progenitor cell release is regulated by circadian rhythm and the peak of CAC release is at Zeitgeber time (ZT)-3 or ZT-5 in non-diabetic rats or mice, respectively^17,18^. Thus, for CAC analysis, we collected peripheral blood and BM tissues from all the animals at around this time point. Consistent with previous reports^15,19,20^, we observed a significant decrease in CACs (Flk-1^+^/Sca-1^+^/CD34^+^ cells) in mice after 3 months and 6 months of diabetes (Fig. 1a). Interestingly, there is no significant difference in CACs from 9-month-diabetic mice and non-diabetic controls. Yet, the absolute percentage of CACs was drastically reduced compared to younger mice, suggesting a potential effect of aging on CACs (Fig. 1a).

While reduced CACs may suggest impaired mobilization of progenitors, it is also possible that the production of progenitor cells is curtailed in the BM. To test this possibility, we quantified BM hematopoietic stem cells (HSCs) and BM-CACs in mice with 1, 3, 6, or 9 months of diabetes and age- and sex-matched non-diabetic controls. We found that the number of HSCs in the BM increased by 33% at 3 months after diabetes (Fig. 1b). BM-CACs (Flk-1^+^/Sca-1^+^/CD34^+^ cells) demonstrate a 1.5-fold increase after 3 months of diabetes followed by a twofold decrease after 6 months of diabetes (Fig. 1c). Furthermore, the number of BM-CACs, but not BM-HSCs, declined drastically with age (Fig. 1b, c).

### Increased expression of ER stress markers in BMNCs and c-Kit-expressing BM cells from diabetic mice

**Fig. 2:**
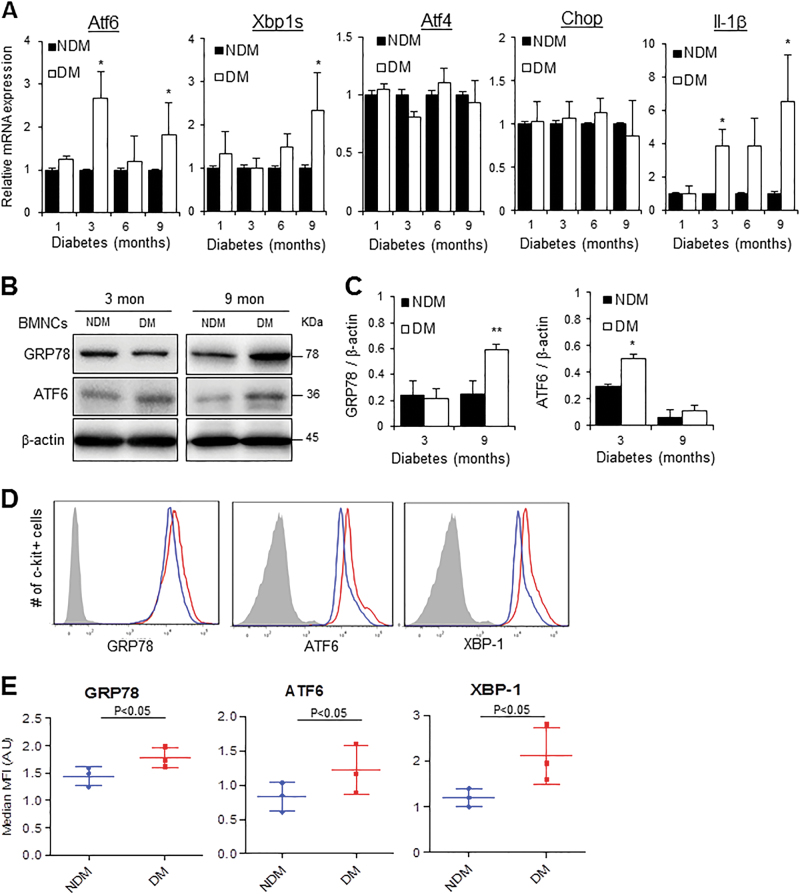
Increased ER stress in BMNCs and c-Kit-expressing BM cells from diabetic mice. Mononuclear cells from the BM of non-diabetic (NDM) and diabetic (DM) mice were separated by Ficoll gradient centrifugation. **a** mRNA levels of ER stress markers (Atf6, Xbp1s, Atf4, Chop) and Il-1β measured by qRT-PCR (*n* = 3 mice per group). **b** Protein levels of ER stress markers in BMNCs measured by western blot analysis. **c** Quantification of protein levels using densitometry (*n* = 3 mice per group; two-way ANOVA with bonferroni post hoc test). **d** Flow cytometric analysis of ER stress in c-Kit-expressing BM cells (represents hematopoietic progenitor cell fraction) from 3-month-diabetic mice. **e** Quantification of median fluorescence intensity (MFI) as compared to NDM control (*n* = 3 mice per group). All data are presented as mean ± SD. *P < 0.05, **P < 0.01 vs. NDM (Student’s *t-*test)

Enhanced ER stress has been observed in EOCs from type 2 diabetic mice and in peripheral blood mononuclear cells (PBMCs) from patients with diabetes and cardiovascular diseases^21–23^. We examined ER stress and inflammation in BM mononuclear cells (BMNCs) of STZ-diabetic mice. We found that the expression of Atf6, a major UPR gene activated in response to ER stress, was upregulated by over twofolds in BMNCs in 3-month-diabetic mice; however, the expressions of Xbp1, Atf4, and Chop were not altered (Fig. 2a). After 6 months of diabetes, both Atf6 and Xbp1 expressions were upregulated in diabetic BMNCs. A similar increase in inflammatory gene IL-1β was observed in BMNCs from 3-month- and 6-month-diabetic mice (Fig. 2a). Western blot analysis shows that the protein level of GRP78, an ER chaperone involved in UPR activation, was increased in BMNCs after 9 months of diabetes (Fig. 2b, c). Interestingly, the protein level of ATF6 was increased after 3 months but not 9 months of diabetes. Since BMNCs is a heterogeneous population of cells consisting of not only hematopoietic cells but many other cell types^24^, we stained BM cells with c-Kit, a marker of the hematopoietic progenitor cells and examined ER stress protein expression in this subpopulation^25^. We found that expression of GRP78, ATF6, and XBP1 was significantly upregulated in BM c-Kit^+^ cells (Fig. 2d, e), suggesting an increase of ER stress in diabetic hematopoietic progenitors.

### Diabetic EOCs display increased ER stress markers

**Fig. 3:**
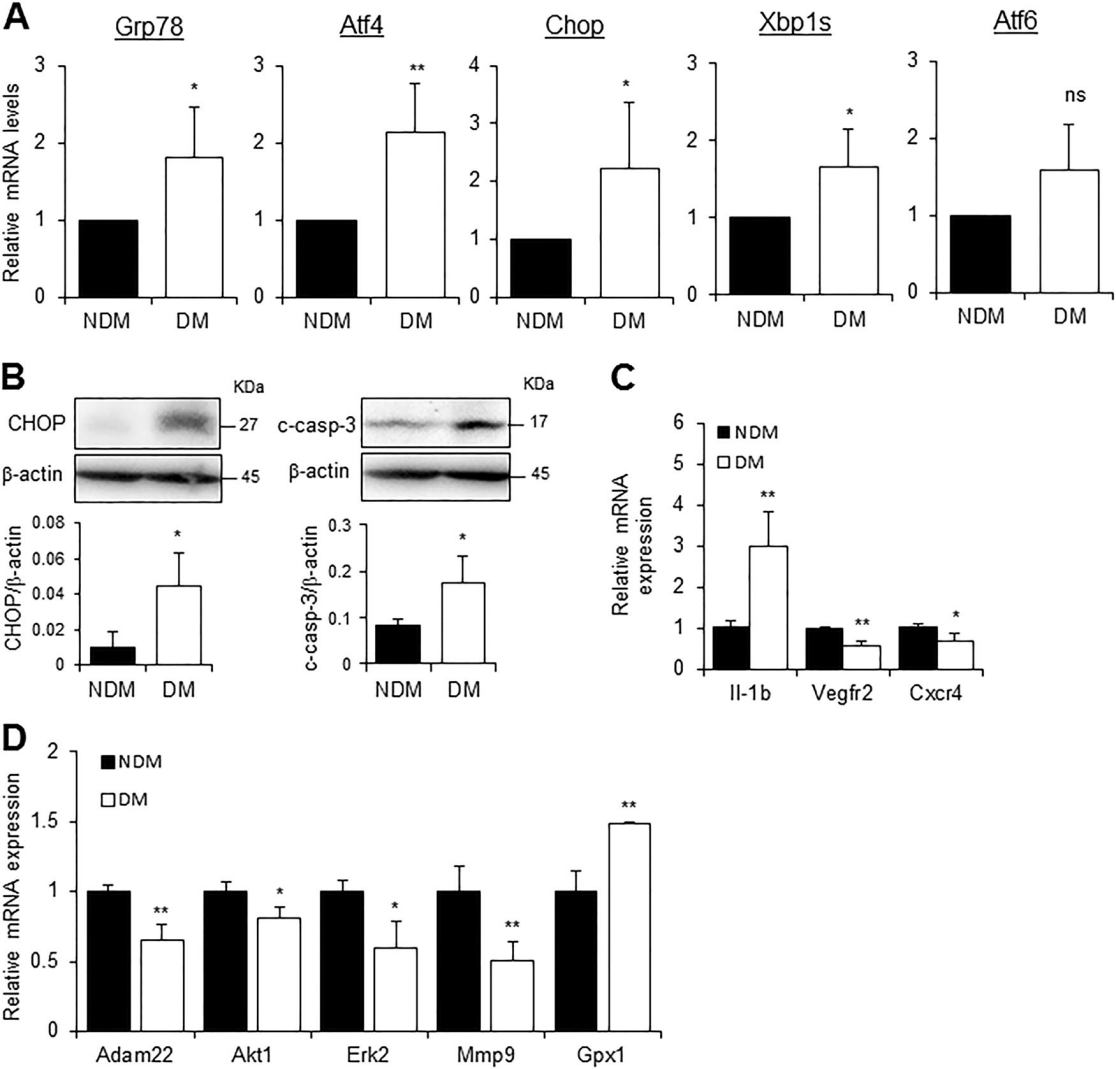
Expression of UPR and functional genes in EOCs. BMNCs from non-diabetic (NDM) and diabetic (DM) mice were cultured in EGM-2 medium for 7 days to generate EOCs. **a** Day 7 EOCs were harvested and expression of ER stress markers was determined by qRT-PCR. Data are presented as mean ± SD (*n* = 4 mice per group). **b** Protein levels of CHOP and cleaved caspase-3 (c-casp-3) in EOCs were examined by western blot analysis and quantified using densitometry (mean ± SD, *n* = 3 mice per group). **c** Expression of ER stress and functional genes in EOCs was measured by qRT-PCR (mean ± SD, *n* = 5–7 mice per group). **d** The mRNA levels of selected UPR and functional genes in EOCs assessed by PCR array (mean ± SD, *n* = 3 mice per group). *P < 0.05, **P < 0.01 (Student’s *t*-test)

BM-derived EOCs are putative angiogenic progenitors, which differentiate from hematopoietic progenitors in vitro. Since hematopoietic progenitors displayed an increase in ER stress, we speculated that EOCs from diabetic mice might also undergo ER stress. As shown in Fig. 3a, there is significant increase in expression levels of Grp78, Atf4, Chop, and Xbp1s in diabetic EOCs as compared to non-diabetic EOCs. Consistent with these changes, the protein levels of CHOP and cleaved caspase-3 were drastically increased (Fig. 3b), suggesting that these cells are undergoing apoptosis. Moreover, the genes responsible for angiogenic function (Vegfr2, Cxcr4, Mmp-9, and Adam22) and cell survival (Akt1 and Erk2) were downregulated (Fig. 3c, d). These defects could further escalate the damage caused by ER stress to diabetic EOCs.

### Inhibition of ER Stress by ex vivo chemical chaperone treatment improves the function of diabetic EOCs

**Fig. 4:**
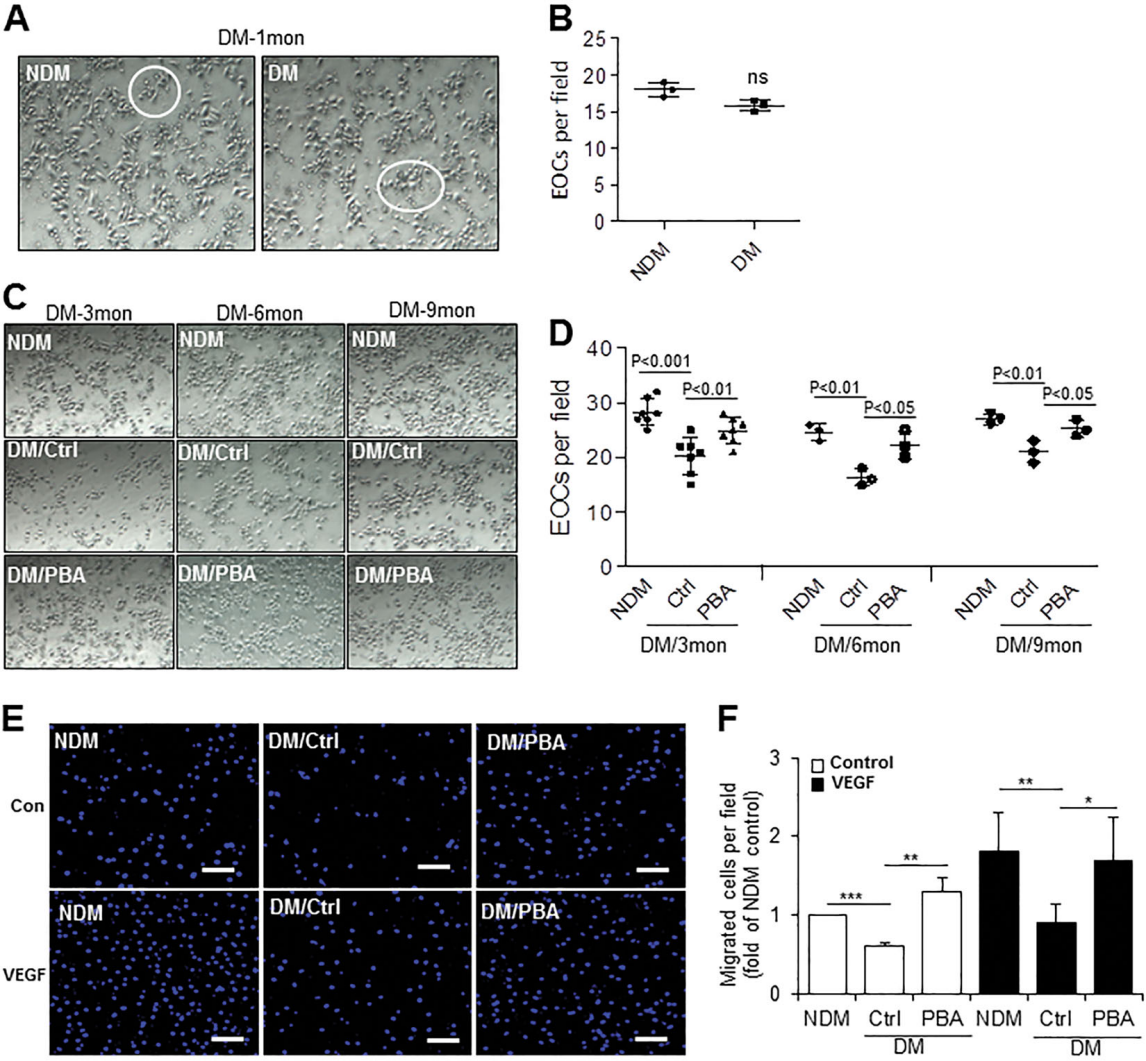
Inhibition of ER stress by ex vivo chemical chaperone treatment enhances the functionality of diabetic EOCs. BMNCs from non-diabetic (NDM) and diabetic (DM) mice were cultured in EGM-2 medium with or without 20 μmol/l of 4-PBA for 7–8 days. **a** EOCs from 1-month-diabetic mice and their non-diabetic controls (white circles) were counted. **b** EOC quantification after 6 days of culture (*n* = 3 mice per group). **c** EOCs were counted. **d** EOC quantification after 6 days of culture (*n* = 3-6 mice per group). **e** In vitro migration assay of EOCs toward VEGF. **f** The migrated cells were stained with DAPI and counted. The results are expressed as fold of NDM control (four mice per group). Scale bars, 100 μm. All data are presented as mean ± SD. *P < 0.05, **P < 0.01, ***P < 0.001, Student’s *t*-test (**a**, **b)** and one-way ANOVA with Tukey’s post hoc test (**c–f**)

To determine whether ER stress plays a role in diabetes-induced angiogenic dysfunction, EOCs isolated from diabetic mice or their age-matched controls were cultured in the presence or absence of chemical chaperone 4-phenobutyrate (4-PBA) for 7–8 days. EOC function was evaluated by counting the number of EOCs per field and migration assay. As shown in Fig. 4a, b, the generation of EOCs was unperturbed after 1 month of diabetes, suggesting that acute hyperglycemia or STZ has no effect on EOC function. In contrast, EOCs from 3- to 9-month diabetic mice displayed markedly impaired EOC generation, which was rescued by 4-PBA treatment (Fig. 4c, d). Because of cell number constraints in mice with longer durations of diabetes, we performed migration assay only in EOCs from 3-month-diabetic mice. As expected, diabetic EOCs failed to migrate toward the chemokine vascular endothelial growth factor (VEGF), and this was almost completely restored by 4-PBA treatment (Fig. 4e, f). These results suggest a critical role of ER stress in diabetes-induced angiogenic progenitor dysfunction.

### Systemic administration of 4-PBA and TUDCA improves CAC release and mitigates BM pathology in diabetic mice

**Fig. 5:**
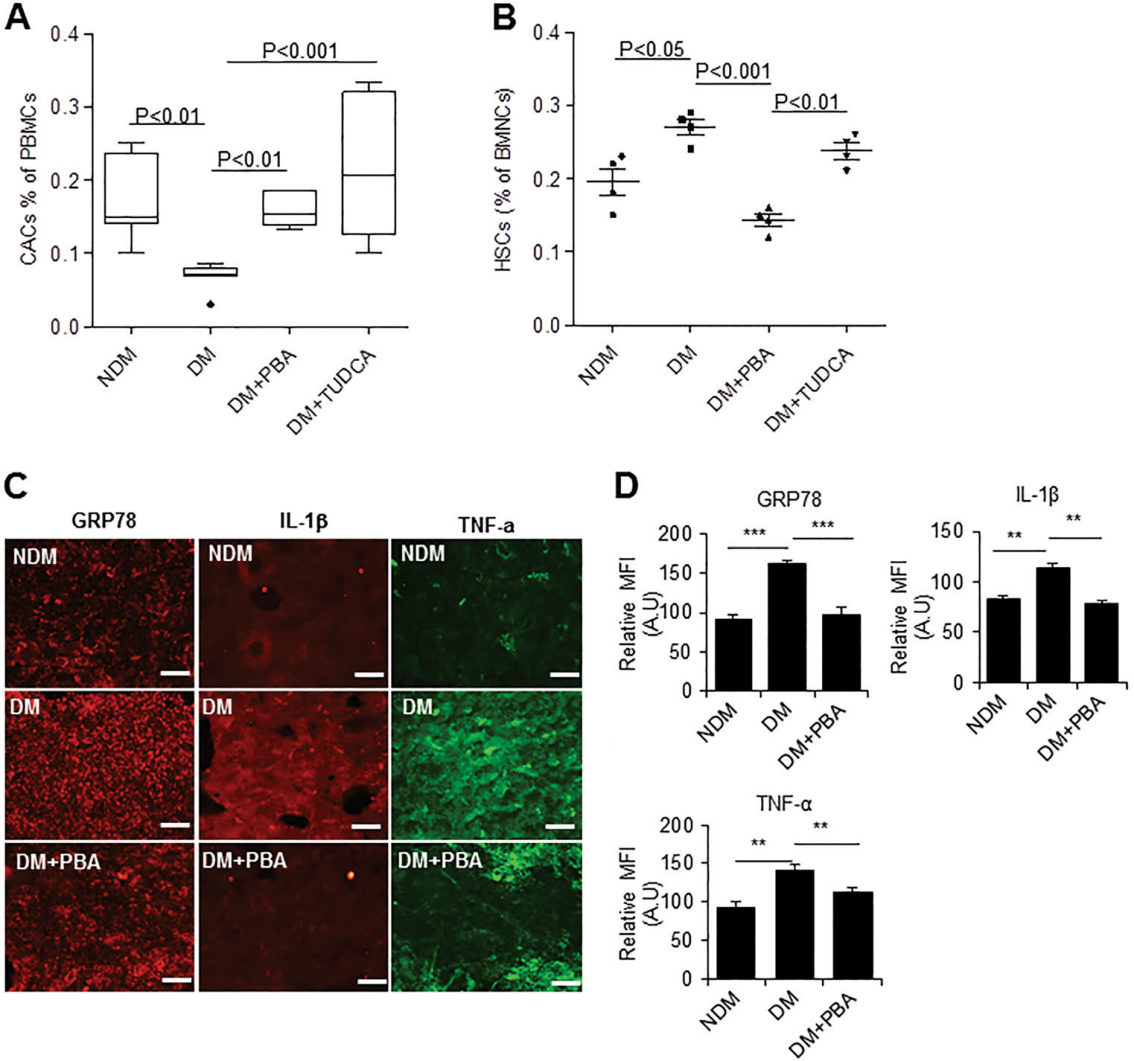
Systemic administration of chemical chaperones improves CAC release and mitigates BM pathology in diabetic mice. Two-month diabetic mice were injected with 40 mg/kg of 4-PBA, 40 mg/kg of TUDCA, or PBS as control, twice a week for 6 weeks. Mononuclear cells from the BM or the peripheral blood were separated by Ficoll gradient centrifugation. **a** Number of peripheral blood CACs (Flk-1^+^/Sca-1^+^/CD34^+^; *n* = 7 mice per group). **b** Number of BM-HSCs (Lin^−^/c-Kit^+^/Sca-1^+^/CD34^+^; *n* = 4 mice per group). **c** Immunohistochemistry showing GRP78 (red), IL-β (red), and TNF-α (green) in the BM sections of non-diabetic, diabetic, and diabetic mice after PBA treatment. **d** Mean fluorescence intensity of GRP78, IL-β, and TNF-α was quantified from four mice per group. Scale bars, 100 μm. All data are presented as mean ± SD. *P < 0.05, **P < 0.01, ***P < 0.001, one-way ANOVA with Tukey’s post hoc test

Mobilization of CACs into the circulation upon vascular injury is critical for vascular repair^26^. To examine the effect of chemical chaperones on CAC release in vivo, 4-PBA and tauroursodeoxycholic acid (TUDCA; 40 mg/kg, twice a week) were administered via intraperitoneal injection in diabetic mice at 6 weeks after diabetes onsets. The dosage was titrated not to alter the blood glucose levels and body weights of the diabetic mice. After 6 weeks of treatment, CAC levels in the peripheral blood were enumerated by flow cytometry. Compared to control mice receiving vehicle treatment, CAC levels were significantly increased in the mice treated with 4-PBA or TUDCA (Fig. 5a). These results suggest that chemical chaperone treatment can improve CAC release from the BM. To determine whether 4-PBA and TUDCA had any effect on HSCs in BM, we quantified the HSCs by flow cytometry. As expected, HSCs were significantly elevated in diabetic mice as compared to non-diabetic controls, which were restored to normal levels mostly with 4-PBA treatment (Fig. 5b).

Next, we determined whether chemical chaperone treatment could alleviate BM pathology. Using immunohistochemistry, we examined ER stress and inflammation markers in BM sections of diabetic and non-diabetic mice. We found that levels of ER chaperone GRP78 and inflammatory markers IL-1β and TNF-α were significantly higher in diabetic BM as compared to non-diabetic control, which were remarkably reduced by 4-PBA treatment (Fig. 5c, d). This suggests that chemical chaperone mitigates BM ER stress and inflammation, potentially improving CAC release.

### Chemical chaperone treatment enhances angiogenic progenitor function and survival in vivo

**Fig. 6:**
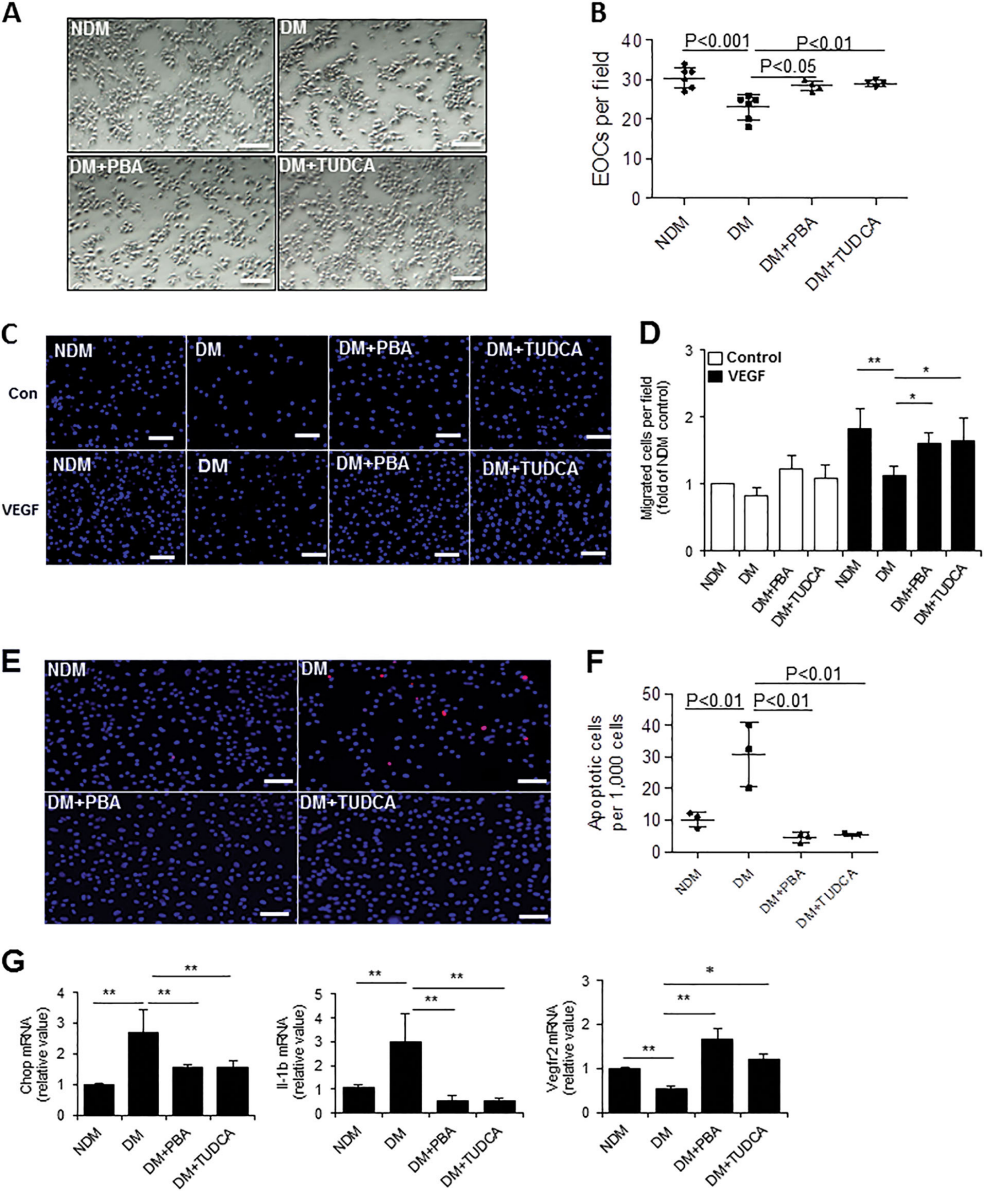
Systemic chemical chaperone treatment enhances the function and survival of angiogenic progenitor cells of diabetic mice. BMNCs from non-diabetic (NDM) and diabetic (DM) after 6-week treatment of 4-PBA, TUDCA, or vehicle were cultured in EGM-2 medium for 7–8 days. **a** The EOCs were counted. **b** EOC quantification after 6 days of culture (*n* = 4–6 mice per group). **c** In vitro migration assay of EOCs toward VEGF. **d** The migrated cells were stained with DAPI and counted and the results were expressed as fold of NDM control (*n* = 4 mice per group). **e** Apoptosis determined by TUNEL assay. **f** Apoptosis was quantified as apoptotic cells per 1000 cells (*n* = 3 mice per group). **g** mRNA levels of Chop, Il-β, and Vegfr2 in EOCs relative to NDM (*n* = 4 mice per group). Scale bars, 100 μm. All data are presented as mean ± SD. *P < 0.05, **P < 0.01, ***P < 0.001 one-way ANOVA with Tukey’s post hoc test

To determine whether systemic chemical chaperone treatment enhances angiogenic function in diabetic mice, EOCs from non-diabetic or diabetic mice after 6-week 4-PBA, TUDCA, or vehicle (PBS) treatment were counted and migration assay was performed. As expected, the generation of EOCs from diabetic mice was reduced and these diabetic EOCs displayed reduced migration toward VEGF. However, both EOC generation as well as their migration toward VEGF was rescued by systemic administration of 4-PBA and TUDCA in diabetic mice (Fig. 6a-d). Furthermore, 4-PBA and TUDCA markedly alleviated diabetes-induced apoptosis of EOCs (Fig. 6e, f).

To elucidate the mechanisms underlying the protective effect of 4-PBA and TUDCA, we examined the expression of genes involved in ER stress, inflammation, and function of EOCs. We found that chemical chaperones significantly downregulated ER stress marker Chop and inflammatory cytokine IL-1β while upregulating Vegfr2 (Fig. 6g). These results suggest that systemic treatment with chemical chaperones significantly improves angiogenic function, reduces inflammation, and enhances their survival.

### 4-PBA protects against retinal vascular degeneration induced by ischemia reperfusion (I/R) injury in diabetic mice

**Fig. 7:**
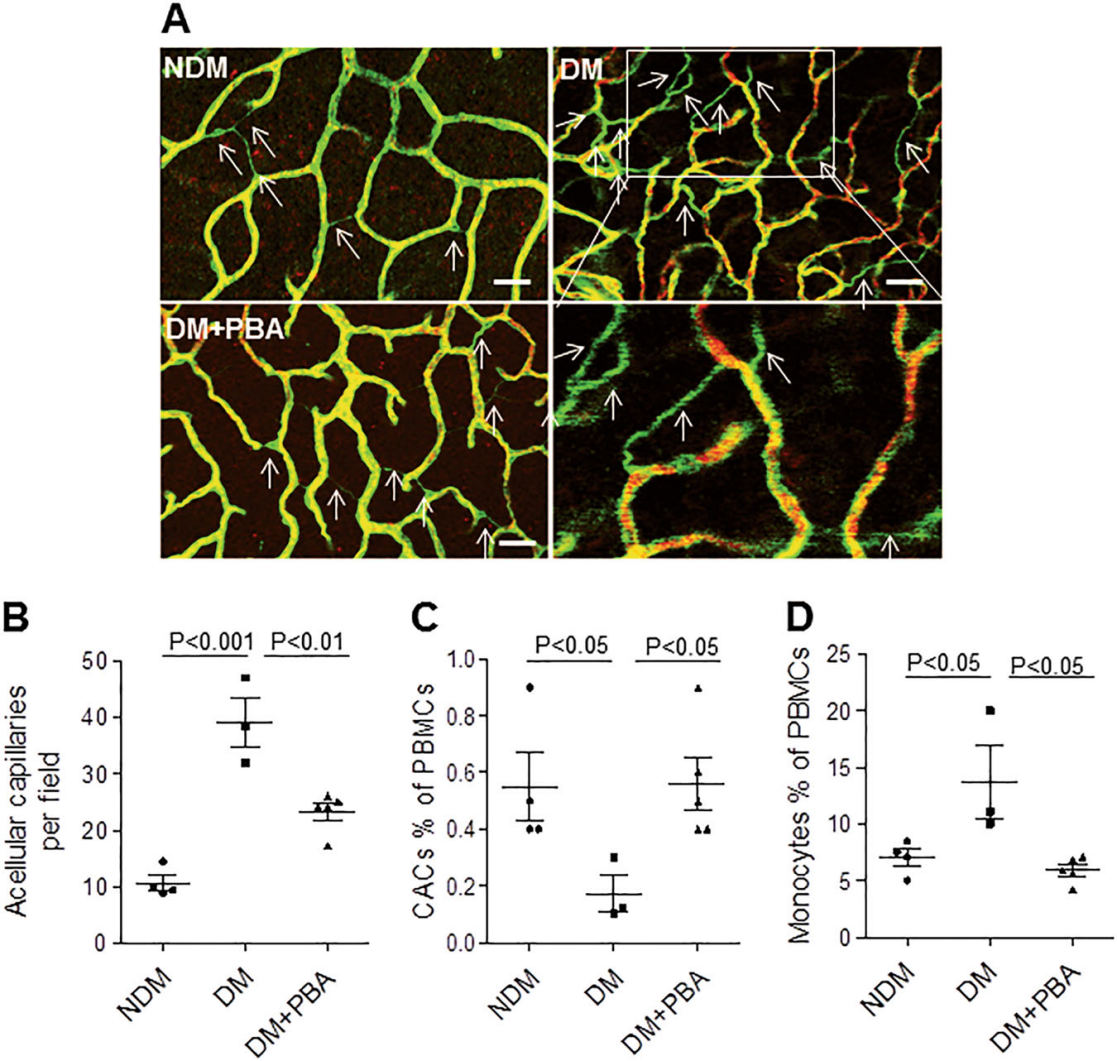
Systemic chemical chaperone treatment mitigates retinal vascular degeneration after I/R in diabetic mice. Retinal I/R was induced in diabetic (DM) mice after 6-week treatment of 4-PBA or vehicle and their non-diabetic (NDM) controls. Retinal vascular degeneration was examined and the numbers of peripheral blood CACs and inflammatory monocytes were determined 14 days after I/R. **a** Representative images of retinal flat mounts stained with Col IV (green) and CD31 (Red). White arrows represent acellular capillaries. Scale bars, 50 μm. **b** Quantification of acellular capillaries per field (*n* = 3–5 mice per group). **c** The number of CACs in the peripheral blood. **d** The number of pro-inflammatory monocytes (CXCR4 + /CD11b + ) in the peripheral blood (*n* = 3–5 mice per group). All data are presented as mean ± SE. *P < 0.05, **P < 0.01, one-way ANOVA with Newman–Keuls multiple comparison

Next, we addressed whether systemic chemical chaperone treatment could mitigate vascular injury induced by retinal ischemia/reperfusion (I/R). This model has been shown to develop retinal acellular capillary formation that recapitulates diabetic vascular changes^27,28^. Six weeks after diabetes onsets, 4-PBA (40 mg/kg, twice a week) was administered intraperitoneally in diabetic mice for 6–8 weeks. At the end of the treatment, retinal I/R was induced in one eye and retinal vascular degeneration was evaluated 14 days following I/R. Acellular capillaries, which are devoid of endothelial cells, are identified as CD31-negative, Col IV-positive vessels. We found that there was a marked increase in acellular capillaries in diabetic mice compared to non-diabetic controls (Fig. 7a, b, white arrows). Treatment with 4-PBA significantly reduced acellular capillary formation following I/R injury in diabetic animals (Fig. 7a,b).

Recent studies suggest that reduced reparative CACs, along with a corresponding increase in pro-inflammatory cells, contribute to insufficient vascular repair^5,29^. Herein, we examined whether the beneficial effect of 4-PBA can be attributed to improved CAC function and/or reduced pro-inflammatory cells by measuring the levels of CACs and monocytes in peripheral blood following I/R injury. Fourteen days post I/R, diabetic mice displayed a marked reduction in CACs and an increase in CXCR4^+^/CD11b^+^ monocytes compared to non-diabetic controls (Fig 7c,d). 4-PBA treatment almost completely restored the numbers of CACs and CXCR4+/CD11b^+^ monocytes (Fig. 7c,d). These results suggest that chemical chaperone improves vascular repair in diabetes possibly by increasing mobilization of CACs and reducing inflammation.

### Knockdown of CHOP improves EOC function and survival under HG conditions

**Fig. 8:**
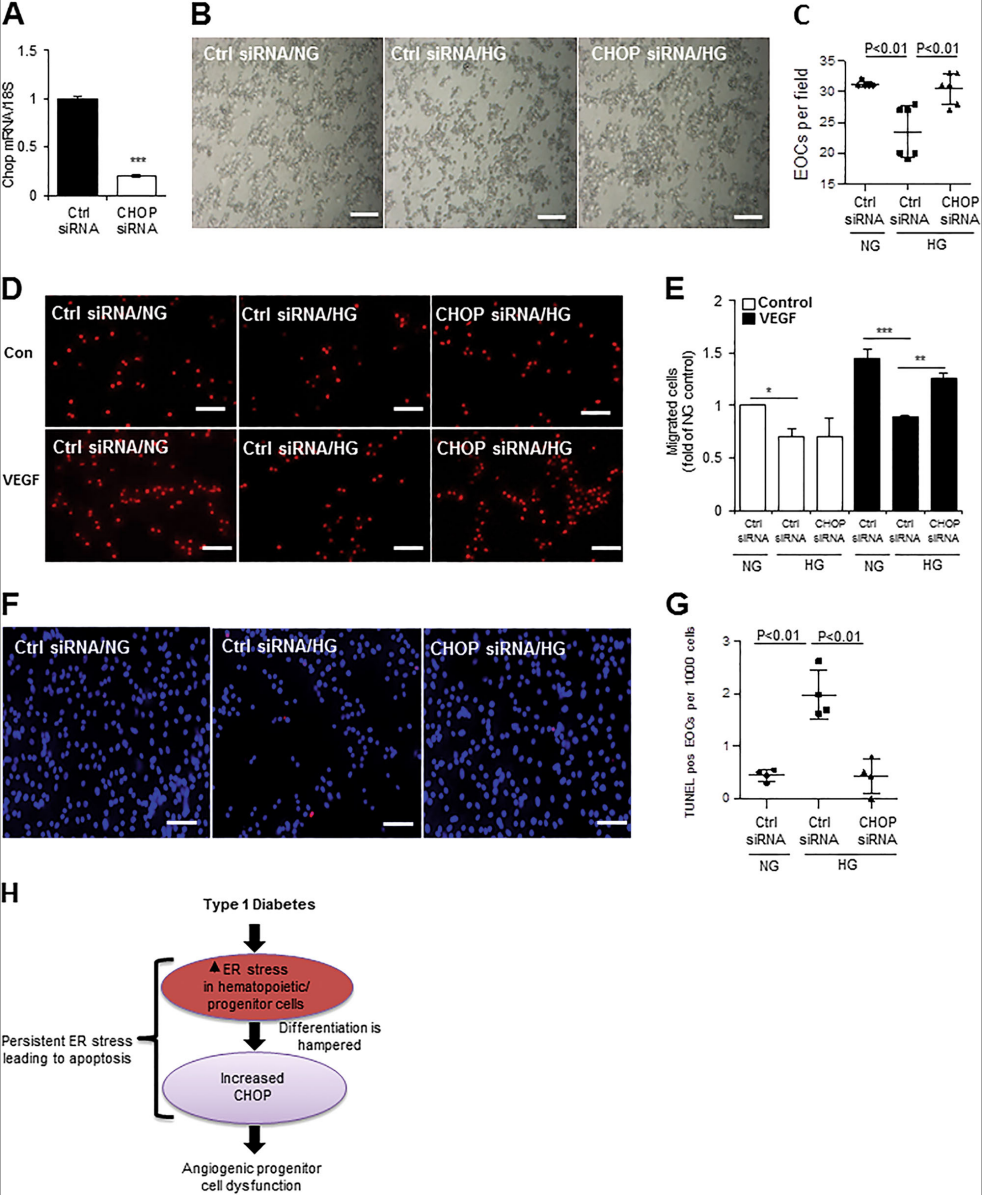
Knockdown of CHOP enhances angiogenic progenitor cell function and survival under HG conditions. BMNCs from C57Bl/6J mice were cultured in EGM-2 medium for 7–8 days. On day 5 or 6, cells were transfected with Chop siRNA for 12 h and then treated with 25 mM HG for 3–4 days. **a** Efficiency of Chop knockdown was evaluated by qRT-PCR. **b** The EOCs were counted and (**c**) quantified after 3–4 days of HG treatment (*n* = 6 mice per group). Scale bars = 100 μm. **d** In vitro migration assay of EOCs toward VEGF. **e** The migrated cells were stained with DAPI and counted. The results are expressed as fold of NG control siRNA (*n* = 3 mice per group). Scale bars = 50 μm. **f** Apoptosis determined by TUNEL assay. **g** It was quantified as apoptotic cells per 1000 cells (*n* = 3 mice per group). Scale bars = 50 μm. **h** Schematic diagram showing activation of UPR pathways in EOCs under type 1 diabetic conditions. In type 1 diabetic conditions hematopoietic progenitor cells are undergoing ER stress, which impedes their differentiation into angiogenic progenitors/EOCs. Persistent ER stress increases ER-induced pro-apoptotic protein CHOP as well as c-caspase-3, suggesting a role for ER stress in EOC dysfunction. All data are presented as mean ± SD. *P < 0.05, **P < 0.01, one-way ANOVA with Tukey’s post hoc test

Our data show an increase in Chop in diabetic EOCs, which was normalized by chemical chaperone treatment. To determine whether CHOP plays a role in diabetic angiogenic progenitor dysfunction, EOCs from C57Bl/6J mice were cultured and transfected with Chop siRNA or control siRNA, and then treated with 25 mmol/l HG for 72 h. Knockdown of Chop was evaluated by qRT-PCR (Fig. 8a). Our results show that HG treatment significantly reduced the generation of EOCs and impaired their migration toward VEGF, both of which were rescued by CHOP knockdown (Fig. 8b-e). TUNEL assay demonstrated that CHOP knockdown protected EOCs from HG-induced apoptosis (Fig. 8f, g). These results suggest for the first time a crucial role for CHOP in angiogenic progenitor dysfunction (Fig. 8h).

## Discussion

It is increasingly evident that dysfunction of angiogenic progenitors is a key factor in vascular complication development^13,17,30^ and poor wound healing in diabetes^31^; yet the mechanisms remain elusive. In current study, we carried out a comprehensive analysis to assess the role of ER stress in BM progenitors using a type 1 diabetes model. We demonstrate a dynamic increase in ER stress accompanied by dysregulated UPR and inflammation in diabetic BM, BMNCs, c-Kit-expressing BM cells, and BM-derived EOCs. Systemic treatment with chemical chaperones leads to a near complete reversal of angiogenic dysfunction, attenuation of BM pathology, and significant enhancement in vascular repair after retinal I/R in diabetic animals. Moreover, CHOP knockdown enhances EOC function and survival under HG conditions. These findings collectively suggest a vital role of ER stress in the pathogenesis of diabetes-induced angiogenic progenitor dysfunction and defects in vascular repair (Fig. 8h).

While a plethora of studies have demonstrated that diabetic CACs are dysfunctional^4,13,32^, a thorough characterization of CACs and BM progenitors, from which CACs differentiate, is lacking. Using the STZ model, we demonstrate a significant increase in BM progenitors after 3 months of diabetes. This is in line with the report by Ferraro et al., which showed an increase in BM-HSCs in 8-week diabetic mice^33^. Similarly, BM-CACs increase after 3 months of diabetes but decrease at 6 months. Although the exact mechanism is unknown, we speculate that the temporary increase in BM-HSCs and CACs could reflect a compensatory response to the early vascular injury in diabetes, while the reduction after 6 months of diabetes may be attributed to impaired BM progenitor differentiation^34^. In contrast to these changes, we observed significant decrease in the numbers of CACs in peripheral blood after 3 months of diabetes. These findings corroborate a previous report demonstrating an inverse correlation between lower number of circulating CACs and the severity of vasculopathy in type 1 diabetic patients^35^, suggesting potential defects in mobilization of CACs from the BM into circulation during diabetes.

While ER stress was found to be increased in EOCs from long-term diabetic *db/db* mice^6^, it remains unknown when ER stress is developed in BM progenitors. We show that ER stress gradually elevates in BMNCs, which is a heterogeneous population of cells comprising progenitor cell/lineage-negative fraction (2–4%) as well as differentiated/lineage-positive cells (96–98%)^36–40^. To assess ER stress only in hematopoietic progenitor cell fraction, we stained BM cells with c-Kit and demonstrated, for the first time, increased ER stress in c-Kit-expressing BM cells in early (3-month) diabetes. These results suggest that hematopoietic progenitors are undergoing ER stress as early as 3 months with diabetes, which therefore may impede their differentiation into CACs (Fig. 8h). Further, 3–9-month diabetic mice exhibited significant reduction in EOC generation (Fig. 4)^19,20^, possibly attributed to hampered differentiation^10,41,42^. To further test this hypothesis, BMNCs were treated with 4-PBA, a chemical chaperone widely used to facilitate protein folding and inhibit ER stress^43^, for the entire duration of culture. Reduction of ER stress significantly improved EOC generation, suggesting a causal role of ER stress in hematopoietic progenitor cell dysfunction in diabetes (Fig. 8h). In addition, reducing ER stress also significantly improves the migration and alleviates apoptosis of EOCs, further confirming the role of ER stress in diabetic angiogenic progenitor dysfunction.

Interestingly, similar to increased ATF4, CHOP, GRP78, and XBP1s activation observed in long-term type 2 diabetic EOCs, we found that transcript levels of these major UPR genes are also upregulated in type 1 diabetic EOCs. The protein levels of ER-induced pro-apoptotic protein CHOP as well as c-caspase-3 are also increased, suggesting that the cells are undergoing apoptosis. These results suggest that both long-term type 2 diabetic EOCs and type 1 diabetic EOCs are undergoing ER stress, specially affecting ATF4/CHOP and IRE1/XBP1 pathways. These cells are more susceptible to apoptosis and display impaired function. Inhibiting ER stress with chemical chaperones successfully restores the function and improves the survival of diabetic EOCs in vitro.

Importantly, our results suggest a beneficial effect of chemical chaperone treatment on improving angiogenic progenitor function in diabetic animals, which is due, at least in part, to the reduction of BM pathologies. Notably, we designed the experiment to avoid the potential effect of chemical chaperones on blood glucose in diabetic mice^8,12^. We show that the number of CACs in the circulation as well as the number of BM progenitors of diabetic mice was almost completely restored by 4-PBA and TUDCA treatment. Moreover, the treatment significantly reduces BM pathologies, a critical factor responsible for impaired function of angiogenic progenitors^44,45^ and impeded mobilization of CACs in diabetes^10,46^. To evaluate whether reducing ER stress in CACs can improve vascular repair in diabetic ischemic retinas, we took advantage of retinal I/R model, which induces vascular degeneration after an acute ischemic injury^28^. Compared to non-diabetic controls, vascular degeneration was escalated in diabetic mice, indicated by higher numbers of acellular capillaries, which was attenuated by 4-PBA treatment. In addition, the number of CACs in peripheral blood was significantly increased while the number of inflammatory monocytes was significantly reduced in 4-PBA-treated mice. These results suggest that alleviating ER stress by chemical chaperones could promote vascular repair by enhancing CAC function. At this point, direct evidence is lacking to demonstrate that chemical chaperone treatment could increase CAC mobilization into injured blood vessels and/or improve the homing capacity of CACs to the BM. This question needs to be addressed in future studies.

One limitation of this study lies in the use of the chemical chaperones 4-PBA and TUDCA, which have been shown to exhibit non-ER-related effects. For example, 4-PBA possesses histone deactetylase (HDAC) inhibitor activity^47^. However, it inhibits HDAC activity at a much higher concentration within the millimolar range. In one report, 4-PBA at a concentration of 20 mmol/l inhibited HDAC activity by ~25%^48^. This concentration is 1000-fold higher than the dose used in our study (20 µmol/l). Therefore, it is unlikely that 4-PBA protects the diabetic EOCs through HDAC inhibition. Moreover, TUDCA, a second chemical chaperone, which does not inhibit HDAC activity, has displayed similar effects to 4-PBA (Figs. 5, 6), thereby suggesting that the beneficial effects are HDAC inhibitor-independent. Future studies to elucidate the mechanisms of the action of these chemical chaperones and to use pharmacological or genetic approaches for UPR manipulation to reduce ER stress in angiogenic progenitor cells are warranted.

Finally, our data indicate that CHOP is a potential molecular mediator of ER stress-associated injury of diabetic angiogenic progenitors. Increased CHOP expression was observed in EOCs isolated from both type 1 (current study) and type 2 diabetic mice^6^. Knockdown of CHOP significantly improved EOC migration and prevented apoptosis induced by HG. However, it remains to be tested whether CHOP inhibition could rescue CAC dysfunction in diabetic animals. The global CHOP knockout mice are readily available and have extensively used in previous studies; however, the roles of CHOP in cell physiology appear to be cell type- and tissue-specific^49,50^. Moreover, the global knockout affects multiple cell types that interact closely with each other, which would make the interpretation of the findings difficult. Thus, the conditional knockout mice that lack CHOP only in angiogenic progenitor cells would help delineate the role of CHOP in diabetes-related CAC dysfunction and vascular degeneration in the future. In summary, our study provides strong evidence that enhanced ER stress and dysregulated UPR is implicated in diabetic BM pathologies and CAC dysfunction, which in turn leads to defective blood vessel repair, ultimately contributing to the development of vascular damage. A better understanding of the exact roles of UPR genes in controlling angiogenic progenitor function could lead to new approach to help prevent and treat diabetic complications.

## Materials and Methods

### Animal studies

Male C57BL/6J mice were purchased from Jackson Laboratory (Bar Harbor, ME, USA). All animal procedures were approved by the Institutional Animal Care and Use Committee at the University at Buffalo, State University of New York. Diabetes was induced by five consecutive intraperitoneal injections of streptozotocin (STZ) at 50 mg/kg/day. Blood glucose levels were measured 1 week after injections and mice with blood glucose greater than 300 mg/dl were considered diabetic. Chemical chaperone treatment was carried out after 6 weeks of diabetes. Briefly, mice were randomly assigned to receive intraperitoneal injection of 4-PBA or TUDCA (Calbiochem, San Diego, CA, USA) at 40 mg/kg or an equivalent amount of vehicle (PBS) twice a week for 6 weeks. Non-diabetic littermates received same vehicle treatment. Blood glucose and body weight were measured every alternate week.

### Flow cytometry-assisted cell sorting analysis

Flow cytometry-assisted cell sorting (FAC) analyses for peripheral blood mononuclear cells (PBMCs) and BM mononuclear cells (BMNCs) were performed using mouse hematopoietic and progenitor cell isolation kit (BD Biosciences, San Diego, CA, USA) as described previously^6^. One million PBMCs and BMNCs were stained with antibodies to label CACs and BM progenitors at 4 °C for 30 min. Flow cytometric analyses were performed with LSRFortessa flow cytometer (BD Biosciences) using Flow-Jo software (Tree Star Inc., Ashland, OR, USA) to delineate CACs (Flk-1^+^/Sca-1^+^/CD34^+^ cells) in peripheral blood, BM-HSCs (Sca-1^+^/c-Kit^+^/CD34^+^/Lin^−^ cells), and BM-CACs (Flk-1^+^/Sca-1^+^/CD34^+^ cells); 100,000 events were counted per sample.

### Early outgrowth cell culture and migration assays

Isolation and culture of EOC were carried out as in our previous study^6^. Briefly, macrophage-depleted mouse BMNCs were plated on six-well plates coated with 5 μg/ml human fibronectin and cultured in endothelial cell basal medium-2 supplemented with 5% FBS, VEGF-A, fibroblast growth factor, insulin-like growth factor-1, epidermal growth factor, ascorbic acid, and antibiotics. Non-adherent cells were removed after 4 days in culture and new medium was applied. The culture was maintained through days 7–9. EOCs, recognized as attaching spindle-shaped cells, were used for further analyses or experiments. EOC counting and Migration assays were performed as described previously^6^.

### TUNEL assay

TUNEL assay was performed using the In Situ Cell Death Detection TMR red kit (Roche Diagnostic, Indianapolis, IN, USA)^6^. Incubation without TdT enzyme was included as a negative control. Cell nuclei were stained with DAPI and TUNEL-positive cells were counted in nine random microscopic fields and results were averaged from three independent experiments.

### Western blotting and qRT-PCR analysis

These assays were performed as previously described^6^. A list of antibodies was provided in Suppl. Table 1 and primer sequences for qRT-PCR can be found in Suppl. Table 2.

### Immunohistochemistry

Mouse bones were fixed in 4% paraformaldehyde for 3 days and then decalcified in 14% EDTA for 2–3 weeks at 4 °C. Following sucrose gradient dehydration process, they were embedded in optimal cutting temperature medium (Sakura Finetek Inc, Torrance, CA, USA) at −80 °C. Cryosections (20 μm) were stained with primary antibodies (Suppl. Table 1) and their corresponding Alexa Flour 488 and Alexa Flour 594-conjugated secondary antibodies, and then examined under an Olympus BX53 microscope (Olympus, Tokyo, Japan).

### Retinal I/R model and quantification of acellular retinal capillaries

Retinal I/R was induced in anesthetized mice^51^. Briefly, the anterior chamber was cannulated with a 30-gauge needle connected to a reservoir of saline. Intraocular pressure (IOP) was adjusted to 80–90 mm Hg to induce retinal ischemia. After 60 min, the cannula was withdrawn to allow reperfusion. Uninjured contralateral eye served as the control. Fourteen days post I/R injury the mouse eyes were enucleated and retinas were prepared for immunofluorescence study of acellular capillaries using antibodies against Col IV and CD31 (Suppl. Table 1). Retinas were imaged under a Zeiss LSM confocal microscope (Carl Zeiss, Oberkochen, Germany). Acellular capillaries were determined as CD31-negative and Col IV-positive vessels, and the number of acellular capillaries were counted and averaged in three to five visual fields per retina.

### PCR array

qRT-PCR array was performed as per the manufacturer’s instruction (SA Biosciences, QIAGEN Inc., Germantown, MD). A complete list of genes was provided in Suppl. Table 3. Results were analyzed using an SA Biosciences online resource RT2 profiler.

### siRNA transfection and HG treatment in EOCs

Transfection of CHOP siRNA (Catalog# 1320001, Thermo Fisher Scientific Inc., Rockford, IL, USA) or control siRNA was performed on day 5–6 EOCs using lipofectamine 3000 as per the manufacturer’s instructions. Transfected cells were treated with normal (5 mM) or high (25 mM) glucose for 72 h and then subjected to functional or apoptosis assays.

### Statistical analysis

Data are expressed as mean ± SD unless noted specifically. Statistical analyses were performed using unpaired Student’s *t-*test for two-group comparisons and one-way ANOVA with Tukey’s post hoc test or Newman–Keuls multiple comparison for three groups or more (GraphPad Prism5, GraphPad Software, San Diego, CA). For the time course experiments, two-way ANOVA with Bonferroni post hoc test was used. Statistical significance was accepted as *P* < 0.05.

## Electronic supplementary material

Supplementary Table 1

Supplmentary Table 2

Supplementary Table 3

Suppl. Fig. 1

Suppl. Fig. 2

Suppl. Fig. 3
