# Supplementary material for: Reduction of Endoplasmic Reticulum Stress Improves Angiogenic Progenitor Cell function in a Mouse Model of Type 1 Diabetes
Source: Cell Death Dis. 2018 Apr 27;9(5):467. doi: 10.1038/s41419-018-0501-5 (PMC5920101; doi:10.1038/s41419-018-0501-5)
Supplement: Supplementary file 1 — Supplementary Table 1 [file 41419_2018_501_MOESM1_ESM.docx]

**Suppl. Table 1. List of antibodies used in western blot analysis (WB), flow cytometry (FC), and immunofluorescence (IF).**

| Antibody | Dilutions | Catalog No. | Company |
| --- | --- | --- | --- |
| anti-GRP78 | 1:1000 (WB) | ab12223 | Abcam |
| anti-GRP78 | 1:50 (FC)  1:100 (IF) | ab21685 | Abcam |
| anti-CREB-2 (C-20) | 1:25 (FC)  1:500 (WB) | sc-200 | Santa Cruz Biotechnology |
| anti-CHOP | 1:500 (WB) | #2895 | Cell Signaling Technology |
| anti-XBP1 | 1:25 (FC)  1:500 (WB) | sc-7160 | Santa Cruz Biotechnology |
| anti-ATF6α | 1:25 (FC) | sc-22799 | Santa Cruz Biotechnology |
| anti-ATF6 | 1:1000 (WB) | ab11909 | Abcam |
| anti-c-caspase-3 | 1:500 (WB) | #9664 | Cell Signaling Technology |
| anti-p-eIF2α | 1:1000 (WB) | #3597 | Cell Signaling Technology |
| anti-β actin | 1:10,000 (WB) | ab8226 | Abcam |
| anti-TNF-α | 1:100 (IF) | sc-52746 | Santa Cruz Biotechnology |
| anti-IL-1β | 1:100 (IF) | AF-401-NA | R&D Systems |
| anti-CD31 | 1:25 | 2h8 | Hybridoma Banks |
| anti-collagen IV | 1:100 | ab6586 | Abcam |
| Alexa Fluor-488 488conjugated | 1:500 | A11034 | Invitrogen |
| Alexa Fluor-594 | 1:500 | 127-585-099 | Jackson Immunoresearch |
