# Supplementary material for: Reduction of Endoplasmic Reticulum Stress Improves Angiogenic Progenitor Cell function in a Mouse Model of Type 1 Diabetes
Source: Cell Death Dis. 2018 Apr 27;9(5):467. doi: 10.1038/s41419-018-0501-5 (PMC5920101; doi:10.1038/s41419-018-0501-5)
Supplement: Supplementary file 2 — Supplmentary Table 2 [file 41419_2018_501_MOESM2_ESM.docx]

| Gene | Primer Sequences |
| --- | --- |
| Mouse Xbp1s | forward, 5’-CCA TCA CAT TGC CTA GAG GAT A-3’ |
|  | reverse, 5’-AGC TGA GTG TCA AAC GAC AAT A-3’ |
| Mouse Chop | forward, 5’- GTC CCT AGC TTG GCT GAC AGA -3’ |
|  | reverse, 5’- TGG AGA GCG AGG GCT TTG -3’ |
| Mouse Atf4 | forward, 5’- CCC CCT TCG ACC AGT CGG GT -3’ |
|  | reverse, 5’- CCG CCT TGT CGC TGG AGA ACC -3’ |
| Mouse Atf6 | forward, 5’- CAG ACT CGT GTT CTT CAA C -3’ |
|  | reverse, 5’- GGC TTC TCT TCC TTC AGT -3’ |
| Mouse Vegfr2 | forward, 5’- TTT GGC AAA TAC AAC CCT TCA GA -3’ |
|  | reverse, 5’- GCA GAA GAT ACT GTC ACC ACC -3’ |
| Mouse Cxcr4 | forward,5’-CCT GTG GCC AAG TTC TTA GTA -3’ |
|  | reverse, 5’-AGG ATC ACG GCT AGC TTT ATT -3’ |
| Mouse Il-1β | forward, 5’-CAG GCA GGC AGT ATC ACT CA -3’ |
|  | reverse, 5’- GAG GAT GGG CTC TTC TTC AA -3’ |

**Suppl. Table 2. List of primers used in qRT-PCR**
