# Supplementary material for: Reduction of Endoplasmic Reticulum Stress Improves Angiogenic Progenitor Cell function in a Mouse Model of Type 1 Diabetes
Source: Cell Death Dis. 2018 Apr 27;9(5):467. doi: 10.1038/s41419-018-0501-5 (PMC5920101; doi:10.1038/s41419-018-0501-5)
Supplement: Supplementary file 3 — Supplementary Table 3 [file 41419_2018_501_MOESM3_ESM.docx]

**Suppl. Table 3. List of genes examined in PCR-Array**

| Position | Gene Symbol | Assay Catalog |
| --- | --- | --- |
| 1 | Nos3 | [PPM03801A*](http://mars.sabiosciences.com/~ywang/sapims/rt_workprocess.php?catalog=PPM03801A) |
| 2 | Cav1 | [PPM03639A*](http://mars.sabiosciences.com/~ywang/sapims/rt_workprocess.php?catalog=PPM03639A) |
| 3 | Cxcr4 | [PPM03149A*](http://mars.sabiosciences.com/~ywang/sapims/rt_workprocess.php?catalog=PPM03149A) |
| 4 | Hif1a | [PPM03799A*](http://mars.sabiosciences.com/~ywang/sapims/rt_workprocess.php?catalog=PPM03799A) |
| 5 | Itgb2 | [PPM03592A*](http://mars.sabiosciences.com/~ywang/sapims/rt_workprocess.php?catalog=PPM03592A) |
| 6 | Mapk1 | [PPM03571A*](http://mars.sabiosciences.com/~ywang/sapims/rt_workprocess.php?catalog=PPM03571A) |
| 7 | Mapk14 | [PPM03578A*](http://mars.sabiosciences.com/~ywang/sapims/rt_workprocess.php?catalog=PPM03578A) |
| 8 | Ifng | [PPM03121A*](http://mars.sabiosciences.com/~ywang/sapims/rt_workprocess.php?catalog=PPM03121A) |
| 9 | Il1b | [PPM03109A*](http://mars.sabiosciences.com/~ywang/sapims/rt_workprocess.php?catalog=PPM03109A) |
| 10 | Il6 | [PPM03015A*](http://mars.sabiosciences.com/~ywang/sapims/rt_workprocess.php?catalog=PPM03015A) |
| 11 | Tgfb1 | [PPM02991A*](http://mars.sabiosciences.com/~ywang/sapims/rt_workprocess.php?catalog=PPM02991A) |
| 12 | Tnf | [PPM03113A*](http://mars.sabiosciences.com/~ywang/sapims/rt_workprocess.php?catalog=PPM03113A) |
| 13 | Ccl2 | [PPM03151A*](http://mars.sabiosciences.com/~ywang/sapims/rt_workprocess.php?catalog=PPM03151A) |
| 14 | Vcam1 | [PPM03208A*](http://mars.sabiosciences.com/~ywang/sapims/rt_workprocess.php?catalog=PPM03208A) |
| 15 | Sele | [PPM03195A*](http://mars.sabiosciences.com/~ywang/sapims/rt_workprocess.php?catalog=PPM03195A) |
| 16 | Csf3 | [PPM02989A*](http://mars.sabiosciences.com/~ywang/sapims/rt_workprocess.php?catalog=PPM02989A) |
| 17 | Fgf2 | [PPM03040A*](http://mars.sabiosciences.com/~ywang/sapims/rt_workprocess.php?catalog=PPM03040A) |
| 18 | Vegfa | [PPM03041A*](http://mars.sabiosciences.com/~ywang/sapims/rt_workprocess.php?catalog=PPM03041A) |
| 19 | Mmp9 | [PPM03661A*](http://mars.sabiosciences.com/~ywang/sapims/rt_workprocess.php?catalog=PPM03661A) |
| 20 | Mmp2 | [PPM03642A*](http://mars.sabiosciences.com/~ywang/sapims/rt_workprocess.php?catalog=PPM03642A) |
| 21 | Kdr | [PPM03057A*](http://mars.sabiosciences.com/~ywang/sapims/rt_workprocess.php?catalog=PPM03057A) |
| 22 | Rhob | [PPM24564A*](http://mars.sabiosciences.com/~ywang/sapims/rt_workprocess.php?catalog=PPM24564A) |
| 23 | Runx3 | [PPM34867A*](http://mars.sabiosciences.com/~ywang/sapims/rt_workprocess.php?catalog=PPM34867A) |
| 24 | Shh | [PPM04516A*](http://mars.sabiosciences.com/~ywang/sapims/rt_workprocess.php?catalog=PPM04516A) |
| 25 | Sox18 | [PPM04494A*](http://mars.sabiosciences.com/~ywang/sapims/rt_workprocess.php?catalog=PPM04494A) |
| 26 | Angpt2 | [PPM03729A*](http://mars.sabiosciences.com/~ywang/sapims/rt_workprocess.php?catalog=PPM03729A) |
| 27 | Col18a1 | [PPM03715A*](http://mars.sabiosciences.com/~ywang/sapims/rt_workprocess.php?catalog=PPM03715A) |
| 28 | Col4a3 | [PPM05147A*](http://mars.sabiosciences.com/~ywang/sapims/rt_workprocess.php?catalog=PPM05147A) |
| 29 | Fn1 | [PPM03786A*](http://mars.sabiosciences.com/~ywang/sapims/rt_workprocess.php?catalog=PPM03786A) |
| 30 | Serpine1 | [PPM03093A*](http://mars.sabiosciences.com/~ywang/sapims/rt_workprocess.php?catalog=PPM03093A) |
| 31 | Timp2 | [PPM03614A*](http://mars.sabiosciences.com/~ywang/sapims/rt_workprocess.php?catalog=PPM03614A) |
| 32 | Timp4 | [PPM03654A*](http://mars.sabiosciences.com/~ywang/sapims/rt_workprocess.php?catalog=PPM03654A) |
| 33 | Adam17 | [PPM05316A*](http://mars.sabiosciences.com/~ywang/sapims/rt_workprocess.php?catalog=PPM05316A) |
| 34 | Adam22 | [PPM36914A*](http://mars.sabiosciences.com/~ywang/sapims/rt_workprocess.php?catalog=PPM36914A) |
| 35 | Gsk3b | [PPM03380A*](http://mars.sabiosciences.com/~ywang/sapims/rt_workprocess.php?catalog=PPM03380A) |
| 36 | Igf2 | [PPM03655A*](http://mars.sabiosciences.com/~ywang/sapims/rt_workprocess.php?catalog=PPM03655A) |
| 37 | Igf1r | [PPM04714A*](http://mars.sabiosciences.com/~ywang/sapims/rt_workprocess.php?catalog=PPM04714A) |
| 38 | Igfbp1 | [PPM05093A*](http://mars.sabiosciences.com/~ywang/sapims/rt_workprocess.php?catalog=PPM05093A) |
| 39 | Map2k1 | [PPM03565A*](http://mars.sabiosciences.com/~ywang/sapims/rt_workprocess.php?catalog=PPM03565A) |
| 40 | Pik3r1 | [PPM03374A*](http://mars.sabiosciences.com/~ywang/sapims/rt_workprocess.php?catalog=PPM03374A) |
| 41 | Pparg | [PPM05108A*](http://mars.sabiosciences.com/~ywang/sapims/rt_workprocess.php?catalog=PPM05108A) |
| 42 | Egfr | [PPM03714A*](http://mars.sabiosciences.com/~ywang/sapims/rt_workprocess.php?catalog=PPM03714A) |
| 43 | Akt1 | [PPM03377A*](http://mars.sabiosciences.com/~ywang/sapims/rt_workprocess.php?catalog=PPM03377A) |
| 44 | Bad | [PPM02916A*](http://mars.sabiosciences.com/~ywang/sapims/rt_workprocess.php?catalog=PPM02916A) |
| 45 | Bcl2 | [PPM02918A*](http://mars.sabiosciences.com/~ywang/sapims/rt_workprocess.php?catalog=PPM02918A) |
| 46 | Casp3 | [PPM02922A*](http://mars.sabiosciences.com/~ywang/sapims/rt_workprocess.php?catalog=PPM02922A) |
| 47 | Casp9 | [PPM03383A*](http://mars.sabiosciences.com/~ywang/sapims/rt_workprocess.php?catalog=PPM03383A) |
| 48 | Foxo3 | [PPM03393A*](http://mars.sabiosciences.com/~ywang/sapims/rt_workprocess.php?catalog=PPM03393A) |
| 49 | Il2 | [PPM02937A*](http://mars.sabiosciences.com/~ywang/sapims/rt_workprocess.php?catalog=PPM02937A) |
| 50 | Prkca | [PPM03501A*](http://mars.sabiosciences.com/~ywang/sapims/rt_workprocess.php?catalog=PPM03501A) |
| 51 | Pten | [PPM03379A*](http://mars.sabiosciences.com/~ywang/sapims/rt_workprocess.php?catalog=PPM03379A) |
| 52 | Raf1 | [PPM03707A*](http://mars.sabiosciences.com/~ywang/sapims/rt_workprocess.php?catalog=PPM03707A) |
| 53 | Stat1 | [PPM04025A*](http://mars.sabiosciences.com/~ywang/sapims/rt_workprocess.php?catalog=PPM04025A) |
| 54 | Trp53 | [PPM02931A*](http://mars.sabiosciences.com/~ywang/sapims/rt_workprocess.php?catalog=PPM02931A) |
| 55 | Bax | [PPM02917A*](http://mars.sabiosciences.com/~ywang/sapims/rt_workprocess.php?catalog=PPM02917A) |
| 56 | Mapk10 | [PPM04539A*](http://mars.sabiosciences.com/~ywang/sapims/rt_workprocess.php?catalog=PPM04539A) |
| 57 | Mapk8 | [PPM03234A*](http://mars.sabiosciences.com/~ywang/sapims/rt_workprocess.php?catalog=PPM03234A) |
| 58 | Mapk9 | [PPM03584A*](http://mars.sabiosciences.com/~ywang/sapims/rt_workprocess.php?catalog=PPM03584A) |
| 59 | Fas | [PPM03705A*](http://mars.sabiosciences.com/~ywang/sapims/rt_workprocess.php?catalog=PPM03705A) |
| 60 | Prdx1 | [PPM04383A*](http://mars.sabiosciences.com/~ywang/sapims/rt_workprocess.php?catalog=PPM04383A) |
| 61 | Prdx6 | [PPM04531A*](http://mars.sabiosciences.com/~ywang/sapims/rt_workprocess.php?catalog=PPM04531A) |
| 62 | Sod2 | [PPM04371A*](http://mars.sabiosciences.com/~ywang/sapims/rt_workprocess.php?catalog=PPM04371A) |
| 63 | Gpx1 | [PPM04345A*](http://mars.sabiosciences.com/~ywang/sapims/rt_workprocess.php?catalog=PPM04345A) |
| 64 | Nfe2l2 | [PPM24614A*](http://mars.sabiosciences.com/~ywang/sapims/rt_workprocess.php?catalog=PPM24614A) |
| 65 | Nox4 | [PPM27908A*](http://mars.sabiosciences.com/~ywang/sapims/rt_workprocess.php?catalog=PPM27908A) |
| 66 | Noxo1 | [PPM36220A*](http://mars.sabiosciences.com/~ywang/sapims/rt_workprocess.php?catalog=PPM36220A) |
| 67 | Duox1 | [PPM30752A*](http://mars.sabiosciences.com/~ywang/sapims/rt_workprocess.php?catalog=PPM30752A) |
| 68 | Txnrd2 | [PPM06198A*](http://mars.sabiosciences.com/~ywang/sapims/rt_workprocess.php?catalog=PPM06198A) |
| 69 | Lpo | [PPM28778A*](http://mars.sabiosciences.com/~ywang/sapims/rt_workprocess.php?catalog=PPM28778A) |
| 70 | Calr | [PPM05020A*](http://mars.sabiosciences.com/~ywang/sapims/rt_workprocess.php?catalog=PPM05020A) |
| 71 | Canx | [PPM34969A*](http://mars.sabiosciences.com/~ywang/sapims/rt_workprocess.php?catalog=PPM34969A) |
| 72 | Dnajb2 | [PPM34963A*](http://mars.sabiosciences.com/~ywang/sapims/rt_workprocess.php?catalog=PPM34963A) |
| 73 | Dnajb9 | [PPM57635A*](http://mars.sabiosciences.com/~ywang/sapims/rt_workprocess.php?catalog=PPM57635A) |
| 74 | Dnajc10 | [PPM26214A*](http://mars.sabiosciences.com/~ywang/sapims/rt_workprocess.php?catalog=PPM26214A) |
| 75 | Dnajc4 | [PPM25617A*](http://mars.sabiosciences.com/~ywang/sapims/rt_workprocess.php?catalog=PPM25617A) |
| 76 | Uggt1 | [PPM35828A*](http://mars.sabiosciences.com/~ywang/sapims/rt_workprocess.php?catalog=PPM35828A) |
| 77 | Edem1 | [PPM26189A*](http://mars.sabiosciences.com/~ywang/sapims/rt_workprocess.php?catalog=PPM26189A) |
| 78 | Edem3 | [PPM40707A*](http://mars.sabiosciences.com/~ywang/sapims/rt_workprocess.php?catalog=PPM40707A) |
| 79 | Erp44 | [PPM35243A*](http://mars.sabiosciences.com/~ywang/sapims/rt_workprocess.php?catalog=PPM35243A) |
| 80 | Eif2a | [PPM26191A*](http://mars.sabiosciences.com/~ywang/sapims/rt_workprocess.php?catalog=PPM26191A) |
| 81 | Eif2ak3 | [PPM26428A*](http://mars.sabiosciences.com/~ywang/sapims/rt_workprocess.php?catalog=PPM26428A) |
| 82 | Atf4 | [PPM04670A*](http://mars.sabiosciences.com/~ywang/sapims/rt_workprocess.php?catalog=PPM04670A) |
| 83 | Atf6 | [PPM33057A*](http://mars.sabiosciences.com/~ywang/sapims/rt_workprocess.php?catalog=PPM33057A) |
| 84 | Creb3 | [PPM25687A*](http://mars.sabiosciences.com/~ywang/sapims/rt_workprocess.php?catalog=PPM25687A) |
| 85 | Ddit3 | [PPM03736A*](http://mars.sabiosciences.com/~ywang/sapims/rt_workprocess.php?catalog=PPM03736A) |
| 86 | Ern1 | [PPM36937A*](http://mars.sabiosciences.com/~ywang/sapims/rt_workprocess.php?catalog=PPM36937A) |
| 87 | Xbp1 | [PPM05627A*](http://mars.sabiosciences.com/~ywang/sapims/rt_workprocess.php?catalog=PPM05627A) |
| 88 | Ero1l | [PPM36023A*](http://mars.sabiosciences.com/~ywang/sapims/rt_workprocess.php?catalog=PPM36023A) |
| 89 | Pdia3 | [PPM04404A*](http://mars.sabiosciences.com/~ywang/sapims/rt_workprocess.php?catalog=PPM04404A) |
| 90 | Dnajc3 | [PPM25697A*](http://mars.sabiosciences.com/~ywang/sapims/rt_workprocess.php?catalog=PPM25697A) |
| 91 | Hspa5 | [PPM03586A*](http://mars.sabiosciences.com/~ywang/sapims/rt_workprocess.php?catalog=PPM03586A) |
| 92 | Actb | [PPM02945A*](http://mars.sabiosciences.com/~ywang/sapims/rt_workprocess.php?catalog=PPM02945A) |
| 93 | B2m | [PPM03562A*](http://mars.sabiosciences.com/~ywang/sapims/rt_workprocess.php?catalog=PPM03562A) |
| 94 | RTC | [PPX63340A](http://mars.sabiosciences.com/~ywang/sapims/rt_workprocess.php?catalog=PPX63340A) |
| 95 | PPC | [PPX63339A](http://mars.sabiosciences.com/~ywang/sapims/rt_workprocess.php?catalog=PPX63339A) |
| 96 | MGDC | [PPM65836A*](http://mars.sabiosciences.com/~ywang/sapims/rt_workprocess.php?catalog=PPM65836A) |
