# Supplementary material for: Reduction of Endoplasmic Reticulum Stress Improves Angiogenic Progenitor Cell function in a Mouse Model of Type 1 Diabetes
Source: Cell Death Dis. 2018 Apr 27;9(5):467. doi: 10.1038/s41419-018-0501-5 (PMC5920101; doi:10.1038/s41419-018-0501-5)
Supplement: Supplementary file 4 — Suppl. Fig. 1 [file 41419_2018_501_MOESM4_ESM.pptx]

## Slide 1
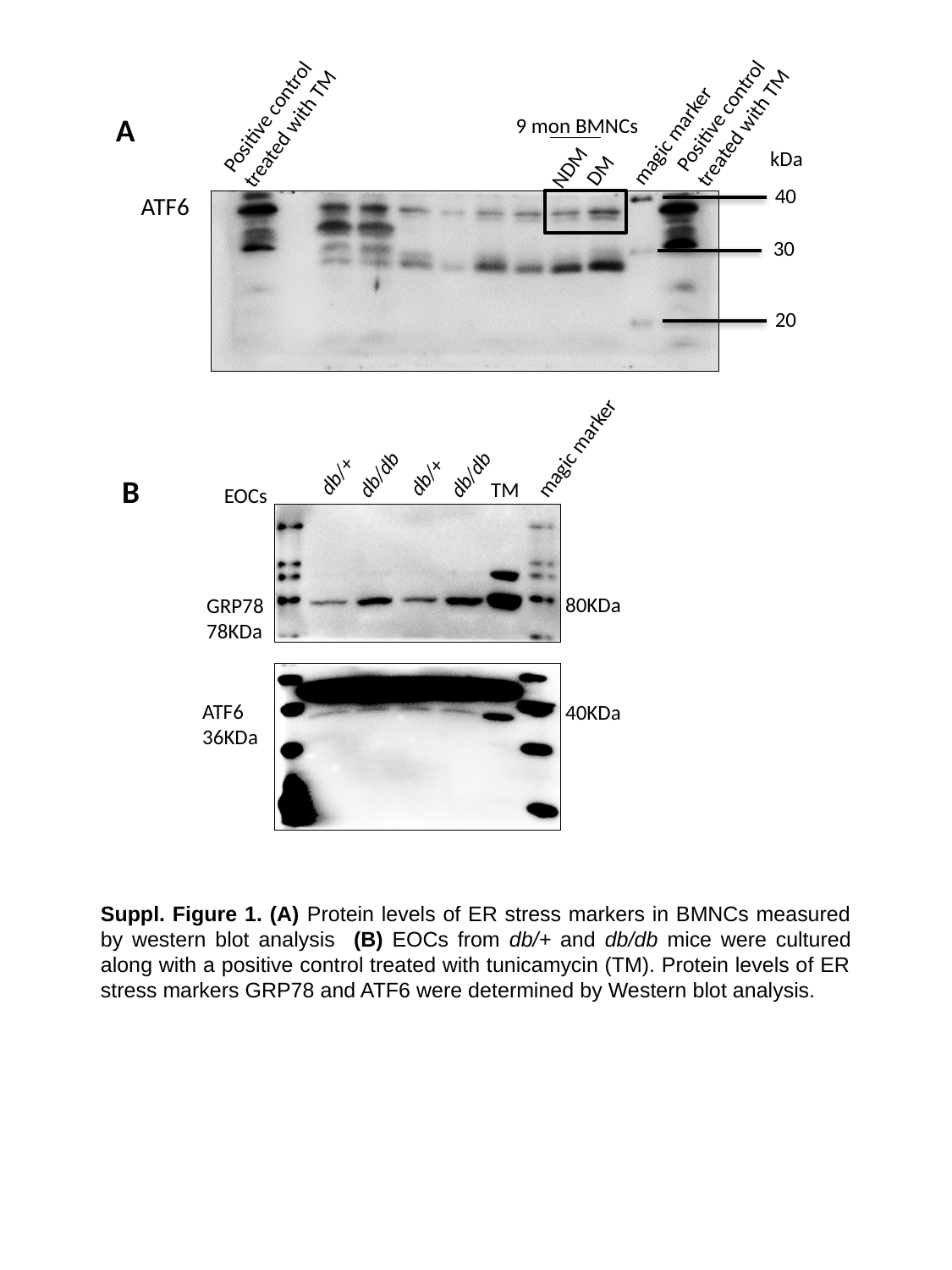

Positive control treated with TM
Positive control treated with TM
A
9 mon BMNCs
magic marker
kDa
NDM
DM
40
ATF6
30
20
magic marker
db/db
db/db
db/+
db/+
B
TM
EOCs
80KDa
GRP78
78KDa
ATF6
36KDa
40KDa
Suppl. Figure 1. (A) Protein levels of ER stress markers in BMNCs measured by western blot analysis (B) EOCs from db/+ and db/db mice were cultured along with a positive control treated with tunicamycin (TM). Protein levels of ER stress markers GRP78 and ATF6 were determined by Western blot analysis.
