# Supplementary material for: Reduction of Endoplasmic Reticulum Stress Improves Angiogenic Progenitor Cell function in a Mouse Model of Type 1 Diabetes
Source: Cell Death Dis. 2018 Apr 27;9(5):467. doi: 10.1038/s41419-018-0501-5 (PMC5920101; doi:10.1038/s41419-018-0501-5)
Supplement: Supplementary file 5 — Suppl. Fig. 2 [file 41419_2018_501_MOESM5_ESM.pptx]

## Slide 1
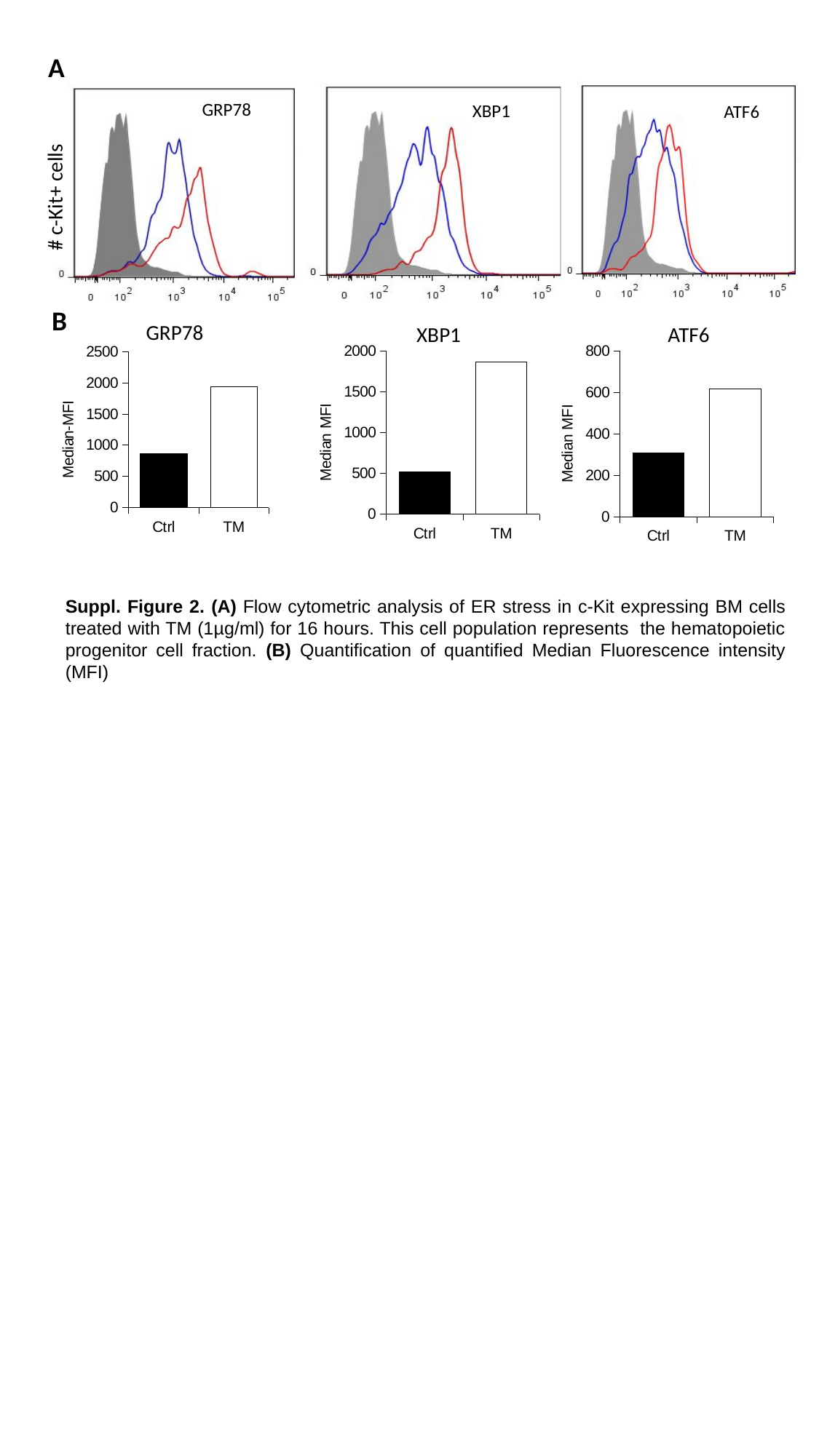

A
GRP78
XBP1
ATF6
# c-Kit+ cells
B
GRP78
XBP1
ATF6
### Chart
| Category | |
|---|---|
| Ctrl | 517.15 |
| TM | 1862.11 |
### Chart
| Category | |
|---|---|
| Ctrl | 309.25 |
| TM | 618.24 |
### Chart
| Category | |
|---|---|
| Ctrl | 868.71 |
| TM | 1938.07 |Suppl. Figure 2. (A) Flow cytometric analysis of ER stress in c-Kit expressing BM cells treated with TM (1µg/ml) for 16 hours. This cell population represents the hematopoietic progenitor cell fraction. (B) Quantification of quantified Median Fluorescence intensity (MFI)
