# Supplementary material for: Reduction of Endoplasmic Reticulum Stress Improves Angiogenic Progenitor Cell function in a Mouse Model of Type 1 Diabetes
Source: Cell Death Dis. 2018 Apr 27;9(5):467. doi: 10.1038/s41419-018-0501-5 (PMC5920101; doi:10.1038/s41419-018-0501-5)
Supplement: Supplementary file 6 — Suppl. Fig. 3 [file 41419_2018_501_MOESM6_ESM.pptx]

## Slide 1
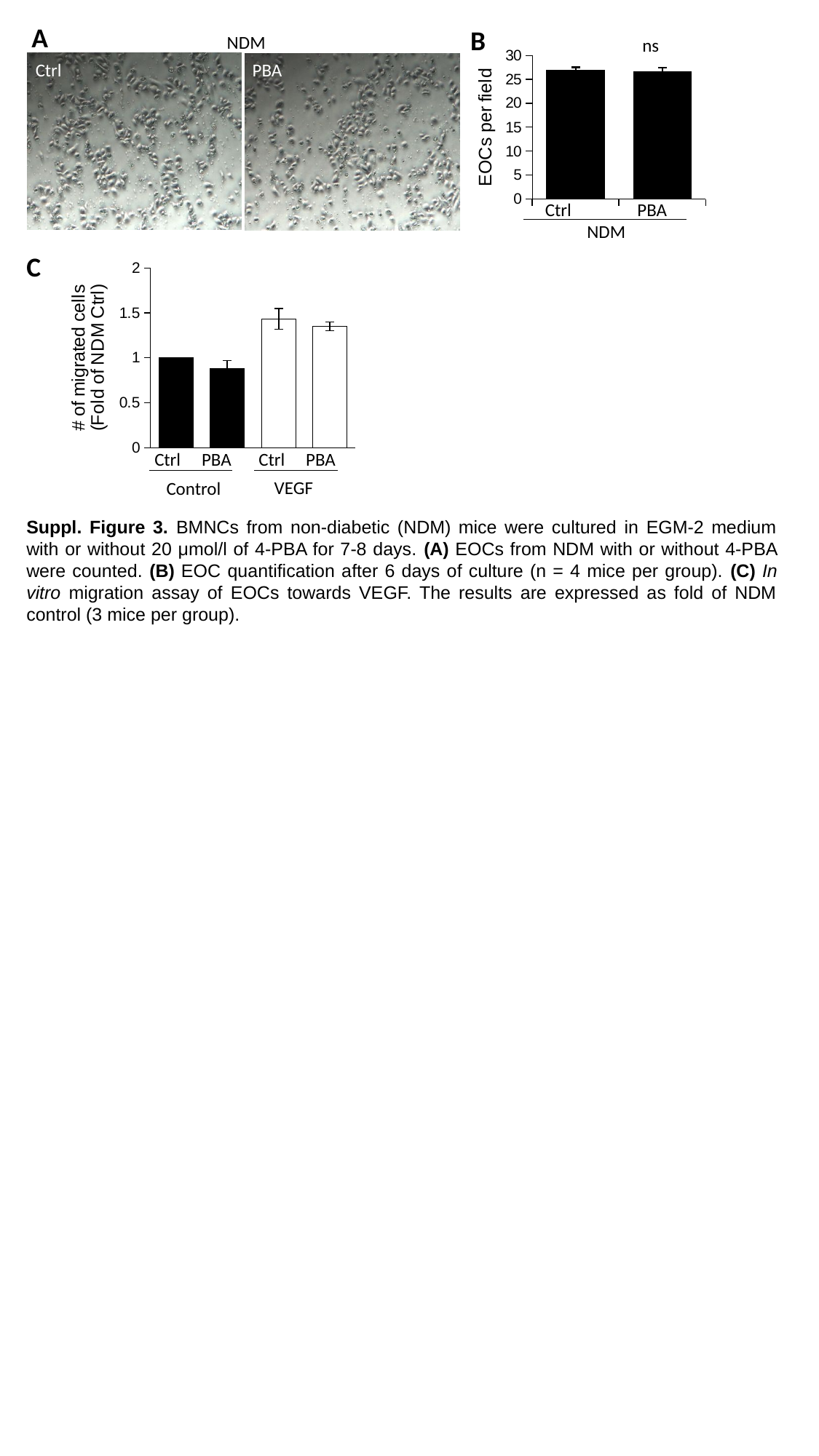

A
B
NDM
Ctrl
PBA
ns
### Chart
| Category | |
|---|---|
| NDM | 26.875 |
| NDM | 26.625 |Ctrl
PBA
NDM
C
### Chart
| Category | |
|---|---|
| NDM | 1.0 |
| NDM+PBA | 0.8766666666666666 |
| NDM | 1.4333333333333333 |
| NDM+PBA | 1.3500000000000003 |Ctrl
PBA
Ctrl
PBA
VEGF
Control
Suppl. Figure 3. BMNCs from non-diabetic (NDM) mice were cultured in EGM-2 medium with or without 20 μmol/l of 4-PBA for 7-8 days. (A) EOCs from NDM with or without 4-PBA were counted. (B) EOC quantification after 6 days of culture (n = 4 mice per group). (C) In vitro migration assay of EOCs towards VEGF. The results are expressed as fold of NDM control (3 mice per group).
